# LncRNA H19 Upregulation Participates in the Response of Glioma Cells to Radiation

**DOI:** 10.1155/2021/1728352

**Published:** 2021-05-31

**Authors:** Yanbei Kuang, Zhitong Bing, Xiaodong Jin, Qiang Li

**Affiliations:** ^1^Institute of Modern Physics, Chinese Academy of Sciences, Lanzhou 730000, China; ^2^Key Laboratory of Heavy Ion Radiation Biology and Medicine of Chinese Academy of Sciences, Lanzhou 730000, China; ^3^Key Laboratory of Basic Research on Heavy Ion Radiation Application in Medicine, Gansu Province, Lanzhou 730000, China; ^4^University of Chinese Academy of Sciences, Beijing 100049, China

## Abstract

Previous studies have indicated that radiation resistance of glioma is one of the leading causes of radiotherapy failure. Mounting evidence suggests that long non-coding RNA (lncRNA) plays an important role in regulating radiosensitivity of cancer cells via implicating in various cell processes. However, the underlying mechanisms remain unclear and need further study, especially at the molecular level. We found that the expression level of lncRNA H19 was elevated by radiation, and then, the modulation of H19 affected the resistant of glioma cells to X-rays. Dual-luciferase reporter analyses showed that H19 was transcriptionally activated by CREB1 in glioma cells after irradiation. In addition, both flow cytometry and 5-ethynyl-2′-deoxyuridine (EdU) assay suggested that H19 was involved in the cell cycle arrest, apoptosis, and DNA synthesis to modulate the radiation response of glioma cells and influenced their radioresistance. Therefore, H19 might play a crucial role in enhancing the radioresistance of glioma.

## 1. Introduction

Glioma is the most prevalent and malignant primary brain tumor in the central nervous system (CNS), and its incidence ranks first among intracranial tumors, accounting for 25.5% of all CNS primary tumors and 80.8% of all CNS malignant tumors [1, 2]. The World Health Organization (WHO) divides gliomas from WHO grade I to WHO grade IV according to the degree of malignancy, among which grade IV glioma is mainly glioblastoma (GBM) [3]. GBM is one of the tumors with a very high mortality rate. Almost all GBM patients receiving treatment will relapse, and the median survival is only about 15 months [4]. Surgery is the principal treatment for glioma patients, in combination with radiotherapy and chemotherapy [5, 6]. Although radiotherapy is an aggressive treatment to shrink tumor volume or prevent tumor relapse after surgical treatment [7, 8], the radiotherapy efficacy is still limited by many factors such as the radioresistance of cancer cells. The radioresistance of glioma is one of the most leading causes of radiotherapy failure.

Emerging evidence has shown that long non-coding RNAs (lncRNAs) more than 200 nt in length play a powerful role in molecular regulation of cancer cells involving in various aspects [9]. H19 is a 2.3-kb lncRNA encoded by the H19 gene located on the chromosome 11p15.5 as an imprinting gene with maternal expression. Many glioma-related studies assumed that H19-derived miR-675 participates in diverse cellular processes including cell invasion [10], proliferation [11, 12], migration [11, 12], cell cycle [11], hypoxia tumor microenvironment [13], and development of glioma [14]. Besides, it has been reported that the H19 expression in glioma tissues is higher than that in para-carcinoma tissues and associated with poor prognosis of glioma patients [15]. Li et al. found that H19 downregulation could enhance the sensitivity of GBM cells to chemotherapy drug temozolomide [16]. Furthermore, acting as a sponge of miRNAs and modulating miRNA action upon target mRNAs is another mechanism by which H19 functions as a modulator. Chen et al. observed that H19 promoted glioma cell proliferation and invasion by sponging of miR-152 [17]. Meanwhile, some researchers hold that H19 plays a key role in regulating the radiosensitivity of cancer cells. H19 promoted the radioresistance of cardiac carcinoma cells via interacting with miR-130a-3p and miR-17-5p [18]. However, another study reported that the suppressed H19 and overexpressed miR-193a-3p level tended to significantly increase the resistance of hepatocellular carcinoma cells to radiation [19]. Few studies have explored whether H19 is involved in the regulation of radioresistance in glioma cells.

cAMP response element binding protein 1 (CREB1) is an important transcription factor belonging to the basic leucine zipper (bZIP) family [20]. It has been found to regulate the transcription of many growth factors and stress response molecules [21]. CREB1 plays essential roles in tumorigenesis via involving in the activation of multitudinous pathway [21, 22]. Researches on the most targets of CREB1 were limited to protein-coding genes [23, 24]; however, the non-coding targets need more exploration.

In this study, we explored whether radiation-induced CREB1 directly activates H19 transcription, and then, the depletion of H19 reduced the radioresistance of glioma cells, aiming at providing a novel target for radiotherapeutic intervention against glioma.

## 2. Materials and Methods

### 2.1. Cells

Human glioma cells lines T98G (WHO grade IV), U87 (WHO grade IV), U251 (WHO grade IV), and human embryonic kidney cell line 293T were acquired from the Chinese Academy of Sciences Cell Resource Centre (Shanghai, China). T98G, U251, and 293T cells were cultured in DMEM containing 10% foetal bovine serum (Bailing Bio, Lanzhou, China), while MEM with 10% foetal bovine serum was used for U87 cell culture.

### 2.2. Irradiation

Irradiation was performed using an X-ray machine (PXi, North Branford, CT, USA) operated at 225 kVp and a dose rate of 2.0 Gy/min at room temperature.

### 2.3. Plasmids and siRNAs

siRNAs (RiboBio, China) and their corresponding negative controls were transiently transfected using riboFECT™ CP Reagent (RiboBio, China) at a final concentration of 50 nM. Each plasmid was transfected by FuGENE® 6 (Promega, USA) at 2.5 *μ*g per 35 mm Petri dish. Overexpressing CREB1 plasmid and its corresponding negative control LentiCMV-IL2-hCD87, pGL3-based construct containing the H19 promoter and its corresponding negative control pGL3, and Renilla luciferase plasmid were purchased from Sangon, Shanghai, China. The target sequences of siRNAs used in this study were as follows:

siRNA-H19: CCTCTAGCTTGGAAATGAA

siRNA-CREB1: GCTCGAGAGTGTCGTAGAA

### 2.4. Bioinformatics Analysis

The Chinese Glioma Genome Atlas (CGGA) and The Cancer Genome Atlas (TCGA) database were adopted to analyse the relationship between the H19 expression and the prognosis of glioma patients.

### 2.5. RNA Extraction and Real-Time PCR

Total RNA was extracted utilizing TRIzol reagent (CWBIO, China). cDNA was obtained via PrimeScript RT Mix reagent (Takara, China). Real-time PCR was conducted with Quantity Nova SYBR Green PCR Master Mix (QIAGEN, Germany), and *β*-actin was used as an internal reference. All procedures were completed in the QuanStudio 5 Real-time PCR system (Therom Lifetech ABI, USA). The primers of H19 were as follows:

Fw: 5′TCCTGAACACCTTAGGCTGG3′

Rev: 5′TGATGTTGGGCTGATGAGGT3′

### 2.6. Dual-Luciferase Reporter Assay

293T cells were cotransfected with the corresponding plasmid according to the experimental design and a Renilla luciferase plasmid as internal reference for 24 h. The reporter activity was tested using the Dual-luciferase-reporter Gene Assay Kit (Beyotime, China).

### 2.7. Colony Formation Assay

After transfection for 24 h, an appropriate number of cells undergoing different transfection were seeded in 6-well plate and placed in an incubator. When the cells adhered to the wall, they were irradiated with X-rays at 0, 1, 2, 4, and 6 Gy. Keeping culture for two weeks, cell colonies were stained with crystal violet for 15 min. Colonies with more than 50 cells were regarded as survivors. Cell survival data were fitted using the linear-quadratic (LQ) model.

### 2.8. Flow Cytometry

Cells were collected at the indicated time points after irradiation. The percentage of apoptosis was detected according to the protocol of Annexin V Apoptosis Detection Kit I (BD Biosciences, USA). The analysis of cell cycle was also completed through flow cytometry. After being harvested and fixed in 70% ice-cold ethanol at -20°C for 48 hours, the cells were stained for 20 minutes on ice with PBS containing 100 *μ*g/mL RNase, 0.2% Triton X-100, and 50 *μ*g/mL PI (Sigma-Aldrich, USA).

### 2.9. 5-Ethynyl-2′-Deoxyuridine (EdU) Assay

DNA synthesis in cells was detected using the Cell-Light EdU DNA Cell Proliferation Kit (RiboBio, China) according to the reagent instructions. Images were taken, and the cells were counted in five randomly chosen visual fields under a microscope (BX51, Olympus, Japan).

### 2.10. Western Blot Analysis

RIPA buffer (Beyotime, China) supplemented with protease inhibitor (Roche, Switzerland) was used to perform the protein extraction. Antibodies against CREB1 (12208-1-AP, Proteintech, China) and *β*-actin (20536-1-AP, Proteintech, China) were purchased for use in the present study.

### 2.11. Statistics

Data are represented as the mean ± standard deviation (SD). One-way ANOVA and Student's *t*-test were performed for comparisons between groups. *p* < 0.05 and *p* < 0.01 were considered statistically significant and statistically extremely significant, respectively.

## 3. Results

### 3.1. Positive Associations between High H19 Expression and Radioresistance of Glioma

**Figure 1:**
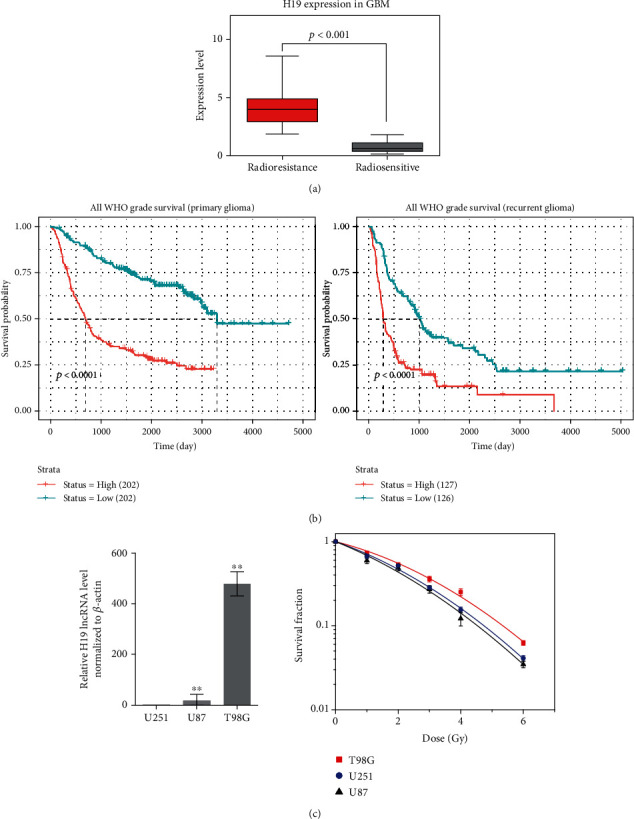
High expression of H19 positively correlates with the radioresistance of glioma. (a) Gene set analysis of H19 expression in patients with GBM who received radiotherapy using the TCGA database. (b) Kaplan-Meier analysis of H19 expression in primary and recurrent glioma patients using the CGGA database. (c) Real-time PCR analysis of H19 expression in T98G, U87 and U251 cells (*n* = 3) and clonogenic survival data of T98G, U87 and U251 cells after irradiation with X-rays (*n* = 3). ^∗^*p* < 0.05; ^∗∗^*p* < 0.01 compared with the control group.

As a first attempt to investigate whether H19 has an impact on the radioresistance of glioma, we first searched for relevant clinical data to analyse their relationship. Shown in Figure 1(a) is the dataset of the Cancer Genome Atlas (TCGA) database. The patients with GBM were divided into two subgroups, radioresistant and radiosensitive, respectively. The H19 expression was significantly higher in the radioresistant patients in contrast to the radiosensitive counterparts. In addition, Kaplan–Meier analysis of the Chinese Glioma Genome Atlas (CGGA) databases indicated a significant relationship between H19 overexpression and primary or recurrent glioma patients' survival rates (Figure 1(b)). These results suggested that the high expression of H19 in patients with GBM was correlated with poor prognosis. To confirm that H19 plays a key role in the radiosensitivity of glioma, we further examined the H19 background level in T98G, U87, and U251 cells using real-time PCR and compared the radioresistance of these cell lines through performing a colony formation assay. As shown in Figure 1(c), in T98G cells, the expression of H19 was several hundred fold higher than those in U87 and U251 cells. As expected, when being compared between cell lines, T98G cell line was the most resistant to radiation while the other two cell lines were more radiosensitive in a similar degree. Collectively, these results suggested a strong correlation between H19 level and the radioresistance of glioma.

### 3.2. Radiation Induced the Expression of H19 and Downregulation of H19 Increased the Radiosensitivity of Glioma Cells

**Figure 2:**
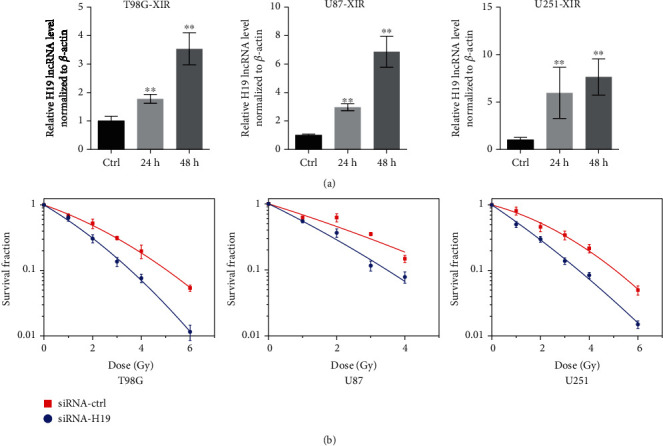
Radiation induced the expression of H19, and downregulation of H19 increased the radiosensitivity of glioma cells. (a) Real-time PCR analysis of the H19 expression in T98G, U87, and U251 cells at 24 h and 48 h postirradiation (*n* = 3). (b) Clonogenic survival data of T98G, U87, and U251 cells transfected with siRNA-ctrl or siRNA-H19 after irradiation with X-rays (*n* = 3). ^∗^*p* < 0.05; ^∗∗^*p* < 0.01 compared with the control group.

According to the aforementioned experimental results, we should investigate whether H19 participates in the radiation response and regulates the radioresistance of glioma cells. Therefore, we harvested cells at different time points after irradiation and observed the changes in the H19 expression. As shown in Figure 2(a), the H19 expression was significantly upregulated in all the three cell lines after irradiation compared to the unirradiated groups. Then, we modified the expression of H19 and observed the changes in the radioresistance of glioma cells. The efficiency of knocking down H19 was shown in SF. As shown in Figure 2(b), H19 downregulation caused a conspicuous increase in the radiosensitivity of glioma cells, and consistent trends were observed in all the three cell lines. Thus, the data demonstrated that the augment of H19 caused by radiation participated in the radiosensitivity regulation of glioma cells and downregulation of H19 increased the radiosensitivity of glioma cells.

### 3.3. Downregulation of H19 Promoted Cell Cycle Arrest and Apoptosis after Irradiation

**Figure 3:**
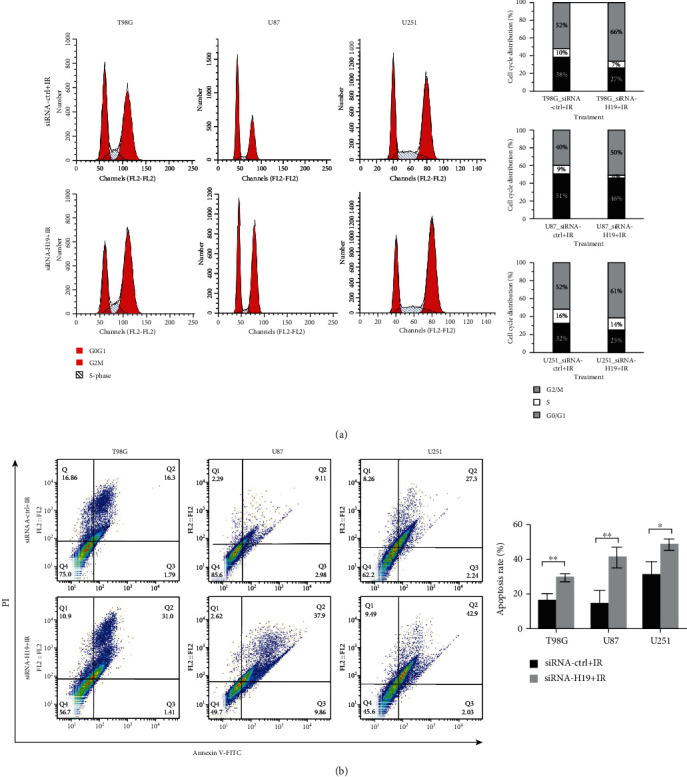
The effects of H19 downregulation on glioma cell cycle and apoptosis after irradiation. (a) Cell cycle distribution in T98G, U87, and U251 cells transfected with siRNA-ctrl or siRNA-H19 at 24 h postirradiation. (b) Cell apoptosis assay in T98G, U87, and U251 cells transfected with siRNA-ctrl or siRNA-H19 at 72 h postirradiation.

The above data prompted us to explore how H19 regulates the radiosensitivity of glioma cells, especially through which pathway. Since irradiation induced the increase of H19, we intended to restrain this upregulation via siRNA and survey the changes in cell cycle and apoptosis. Cycle arrest was detected by flow cytometry at 24 h postirradiation. Compared with the siRNA-control group, an increasing percentage of G2/M phase cells was observed in the siRNA-H19 group of the three cell lines (Figure 3(a)), accompanied by a decrease in the proportion of G0/G1 and S phase cells. Besides, apoptosis rate analysis was conducted at 72 h postirradiation (Figure 3(b)). Our results indicated that cell apoptosis was significantly induced at 72 h after irradiation. The rate of apoptosis was dramatically increased in all siRNA-H19 groups when compared to the respective siRNA-control group in glioma cell lines. These results showed that H19 suppression might regulate the radiosensitivity of glioma cells via enhancing cell cycle arrest and apoptosis.

### 3.4. Downregulation of H19 Reduced DNA Synthesis after Irradiation

**Figure 4:**
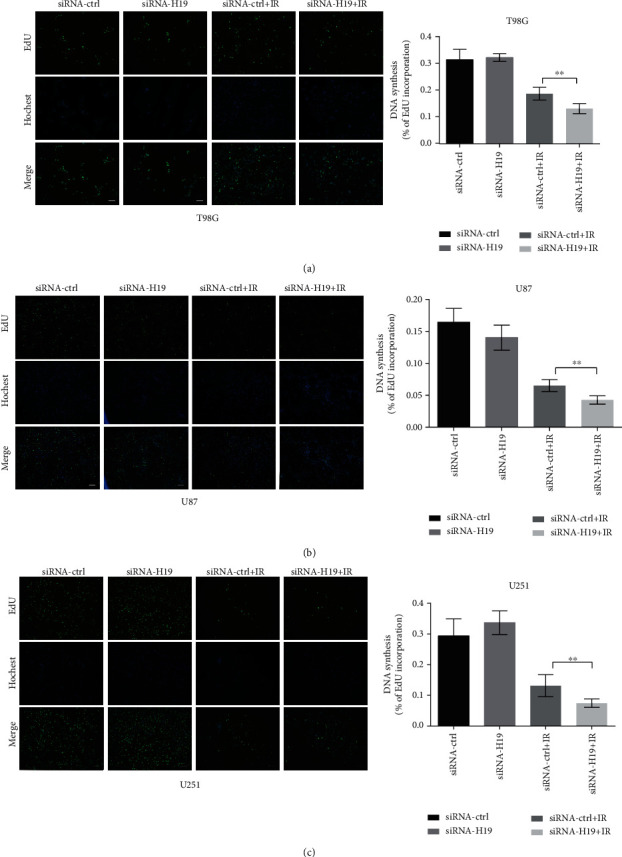
The effects of H19 downregulation on DNA synthesis. (a–c) DNA synthesis analysis of T98G, U87, and U251 cells transfected with siRNA-control or siRNA-H19 at 24 h postirradiation based on the EdU assay (scale bars, 50 *μ*m, *n* = 5). ^∗^*p* < 0.05; ^∗∗^*p* < 0.01 compared with the control group.

DNA synthesis situation was investigated using the EdU assay. As shown in Figures 4(a)–4(c), glioma cells transfected with siRNA-H19 exhibited no differences compared to the siRNA-control group in DNA synthesis. However, when cells were exposed to irradiation, the DNA synthesis obviously decreased in the siRNA-H19 + IR group compared with the siRNA-control+IR group. The evidence from the EdU incorporation assay supported that H19 could modulated the radiosensitivity of glioma cells, because repression of H19 could inhibit DNA replication activity after irradiation.

### 3.5. H19 Was a Direct Transcriptional Target of CREB1

**Figure 5:**
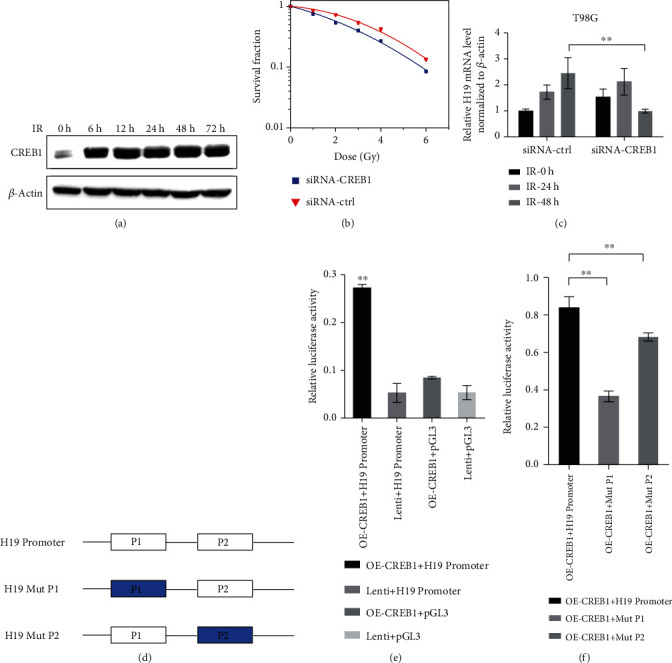
H19 is a direct transcriptional target of CREB1. (a) The expression of CREB1 in T98G cells at different time points after irradiation. (b) Clonogenic survival assay of T98G cells after transfection with siRNA-ctrl or siRNA-CREB1 and irradiation with X-rays (*n* = 3). (c) Real-time PCR analysis of H19 expression in T98G cells transfection with siRNA-ctrl or siRNA-CREB1 and irradiation with X-rays (*n* = 3). (d) Schematic illustration of firefly luciferase reporter plasmids. (e, f) Relative luciferase activity in 293T cells co-transfected with the indicated reporter constructs and Renilla luciferase plasmid (*n* = 3). Lenti is the vector of OE-CREB1 and pGL3 is the vector of H19 Promoter. ^∗^*p* < 0.05; ^∗∗^*p* < 0.01 compared with the control group.

We used the RegRNA2.0 database to predict the proteins which could bind to H19 and found that CREB1 is one of them. Therefore, first we detected the CREB1 expression following irradiation. As shown in Figure 5(a), high expression of CREB1 persisted for several days starting at 6 h after irradiation. Then, we depleted the expression level of CREB1 and found the CREB1 downregulation caused an increase in the radiosensitivity of glioma cells (Figure 5(b)). CREB1 and H19 had a positive correlation relationship in expression and function. Next, we silenced CREB1 and explored the changes of the H19 expression in T98G cells after irradiation. As shown in Figure 5(c), there was no obvious difference in the H19 expression in the siRNA-control and siRNA-CREB1 groups at 24 h postirradiation. However, at 48 h postirradiation, radiation-induced H19 augment was markedly attenuated by transfection with siRNA-CREB1. Therefore, CREB1 downregulation would reduce the H19 expression level after irradiation. It is tempting to speculate that CREB1 could activate the transcription of H19 when cells received irradiation. Hence, we conducted a dual-luciferase reporter assay to investigate whether CREB1 could mediate the H19 expression at the transcriptional level. We examined the genomic promoter regions of H19 in JASPAR. Two potential binding sites named P1 and P2 were found within the promoter of H19 (Figure 5(d)), respectively, located at -1638 to -1627 kb and -1605 to -1594 kb of H19. DNA fragments containing wild-type P1 and P2 sites (H19 promoter) or, respectively, mutational P1 (H19 Mut P1) and P2 (H19 Mut P2) site were cloned into the promoter region of a firefly luciferase reporter plasmid (pGL3). The three generated plasmids (Figure 5(d)) were used in the subsequent experiments. The results, as shown in Figure 5(e), indicated that the luciferase activity of the group cotransfected CREB1 overexpression plasmid (OE-CREB1) and H19 Promoter plasmid is significantly higher than the other control groups, suggesting that the P1 and P2 sites were responsible for the transcription of H19. To confirm this deduction, we transfected Mut P1 or Mut P2 to rescue luciferase expression. As expected, the increase of the OE-CREB1 + H19 promoter group in luciferase expression was markedly attenuated by the mutation of P1 or P2 sites (Figure 5(f)). In addition, the efficiency of siRNA-CREB1 and overexpressing CREB1 was shown in SF. Thus, the data above demonstrated that H19 was transcriptionally activated by CREB1.

## 4. Discussion

This study articulated the upregulation mechanism of H19 in glioma cells exposed to X-rays and indicated that H19 might exert an influence on radiosensitivity of glioma cells through apoptosis, cell cycle arrest, and DNA synthesis.

Previous studies reported that there is a positive correlation between the high expression of H19 and the poor prognosis in glioma patients [15]. The findings from our study (Figure 1(b)) are consistent with the previous results. H19 indeed acts as an enhancer of tumorigenesis and development in glioma. In respect to radiosensitivity, the results of the cell level validation (Figure 1(c)) were consistent with the bioinformatics analysis (Figure 1(a)). The H19 expression in T98G cells was much higher than the other two cell lines, and T98G cells were the most resistant to X-rays. Although H19 level in U87 cells was 18-fold higher than that in U251 cells, they had a similar radiation resistance as shown in Figure 1(c). We suppose that the radioresistance of glioma cells is determined not only by H19 but also by the entire genetic background, but the significant role of H19 cannot be denied.

The increase of H19 after irradiation (Figure 2(a)) indicated that H19 participated in the response of glioma cells to X-rays. Concretely, H19 suppression promoted G2/M arrest and apoptosis compared to the control group (Figure 3). The augment of G2/M arrest means that it took longer time for the siRNA-H19 group to resume cell cycle progression after irradiation compared with the control group, suggesting that DNA damage repair was more severely blocked in the siRNA-H19 group. Besides, the siRNA-H19 group had a higher rate of apoptosis indeed, indicating that more irreparable damages occurred in cells when H19 was downregulated. Nonetheless, the mechanisms underlying cell cycle arrest and apoptosis enhancement by H19 downregulation remain further investigation.

However, we have conducted a preliminary research (data not shown). Our results define p21 acting as a downstream molecule of H19 in glioma cells, and p21 could modulate radiation-caused G2/M arrest and apoptosis [25]. Future studies are needed to refine this point. Of course, there is a considerable body of existing researches regarding the modulation of H19 to cell cycle and apoptosis through other pathways. H19 inhibits the apoptosis process via the downregulation of proapoptotic factor Bax as well as tumor suppressor factor p53 [26]. Besides, H19 contacts with the apoptosis process and cell cycle depending on the regulation to miR-138 and SOX4 [27], miR-675 [28], miRNA-107 [29], YAP1 [30], and Ras/Raf/MEK/ERK cascade [31]. Therefore, the molecular mechanisms underlying the H19 cellular functions are complex.

Regarding the transcriptional activation of H19, the action mechanism for CREB1 has been well-documented in this study. According to Figure 5(f), P1 and P2 both had transcriptional modulatory activity, and P1 site appeared to play a more important role than P2. Furthermore, as shown in Figure 5(c), H19 level in the siRNA-CREB1 groups at 24 h postirradiation was still increased compared to the time point of 0 h after irradiation. This phenomenon can be explained by the fact that other transcriptional factors could also promote the H19 expression according to our results (data not shown). Therefore, the upregulation of H19 postirradiation is of great importance for radiation response in glioma cells.

This study elucidated the mechanism of H19's transcriptional activation after irradiation and identified H19 as a potential target to improve the radiotherapy efficacy of glioma. Apart from these, H19 has the potential to act as a biomarker for the prediction of glioma patients' radiosensitivity.

## 5. Conclusion

In conclusion, the present study verified that radiation-induced CREB1 directly activated H19 transcription and upregulated H19 participated in radioresistance regulation of glioma cells via several cellular process, including apoptosis and G2/M phase arrest and DNA synthesis. Thus, our data indicated that H19 plays a key role in regulating the radiosensitivity of glioma cells.

## Data Availability

The datasets generated during and/or analysed during the current study are available from the corresponding author on reasonable request.

## Supplementary Materials

Supplementary Materials Supplemental Figure: (a) The siRNA effect of H19 was detected at 24 h and 48 h post-transfection using real-time PCR. (b) The siRNA and overexpression effect of CREB1 at the protein level were determined 24 h post-transfection using western blotting. ^∗^*p* < 0.05; ^∗∗^*p* < 0.01 compared with the control group.
