# Supplementary material for: LncRNA H19 Upregulation Participates in the Response of Glioma Cells to Radiation
Source: Biomed Res Int. 2021 May 31;2021:1728352. doi: 10.1155/2021/1728352 (PMC8187074; doi:10.1155/2021/1728352)
Supplement: Supplementary Materials — Supplemental Figure: (a) The siRNA effect of H19 was detected at 24 h and 48 h post-transfection using real-time PCR. (b) The siRNA and overexpression effect of CREB1 at the protein level were determined 24 h post-transfection using western blotting. ∗p < 0.05; ∗∗p < 0.01 compared with the control group. [file 1728352.f1.docx]

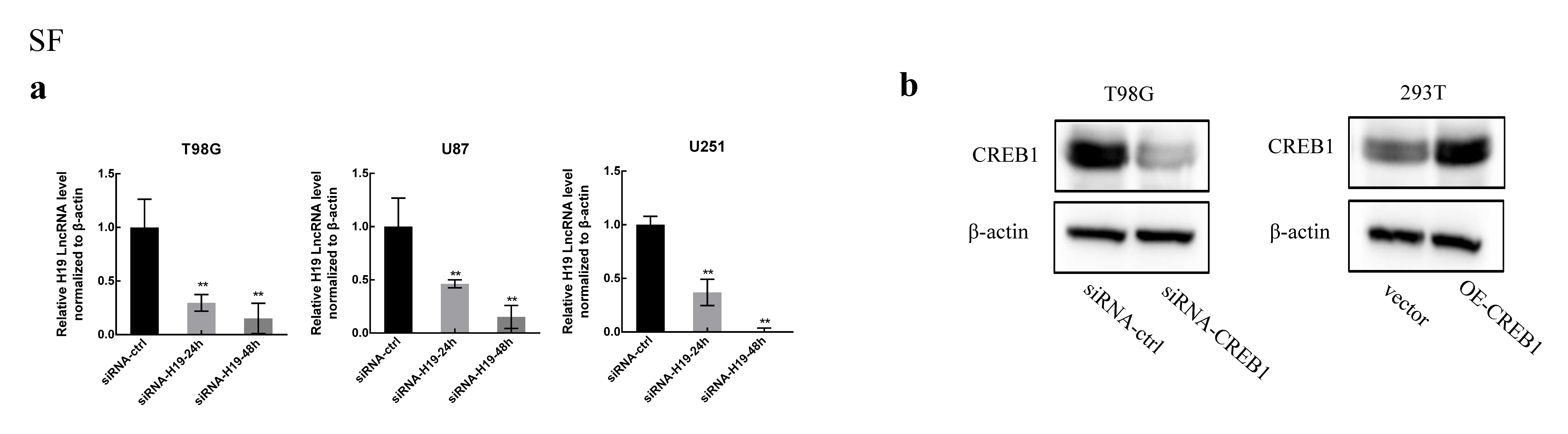


**Supplemental Figure**

**a**: The siRNA effect of H19 was detected at 24 h and 48h post-transfection using real-time PCR. **b**: The siRNA and overexpression effect of CREB1 at the protein level were determined 24 h post-transfection using western blotting. *p < 0.05; **p < 0.01 compared with the control group.
